# Low serum phosphate is not diagnostic for refeeding syndrome in critical care

**DOI:** 10.1186/2197-425X-3-S1-A282

**Published:** 2015-10-01

**Authors:** N Venugopal, P Turner, R Kolamunnage-Dono, C Downey, ID Welters

**Affiliations:** University of Liverpool, Liverpool, United Kingdom; Royal Liverpool University Hospital, Liverpool, United Kingdom; University of Liverpool, Institute of Translational Medicine, Liverpool, United Kingdom; Royal Liverpool University Hospital, Intensive Care Unit, Liverpool, United Kingdom; University of Liverpool, Institute of Ageing and Chronic Disease, Liverpool, United Kingdom

## Introduction

Refeeding syndrome (RFS) is a complication of malnutrition. It consists of electrolyte derangements and organ failure. National Institute of Clinical Excellence (NICE) have outlined specific risk factors for identifying patients at risk of RFS, including low levels of phosphate, potassium and magnesium before feed, low body mass index, starving for >5 days, weight loss, history of alcohol abuse and drugs including insulin, diuretics, chemotherapy and antacids. Hypophosphataemia is a common phenomenon in critically ill patients, but may also indicate RFS. As there are no specific criteria for diagnosing RFS in critical illness, hypophosphataemic patients are often nutritionally restricted to prevent development of RFS.

## Objective

To assess the applicability of the NICE risk factors to diagnose RFS in critically ill patients

## Methods

We analysed hypophosphataemic patients admitted to Intensive Care Unit (ICU) in Royal Liverpool Hospital between January 2010- January 2012. Demographic details, risk factors for RFS, electrolyte values and nutritional details over a 7 day period were collected. Moderate and severe hypophosphataemia were defined as serum phosphate levels between 0.32- 0.65 mmol/L and < 0.32 mmol/L respectively at any time during the observation period. The significant difference between groups were analysed using independent t-Test and Mann-Whitney U test depending on distribution of continuous variables. Categorical data was analysed using χ^2^ test or Fisher's exact test. A receiver operative characteristic (ROC) curve was plotted to analyse the predictability of RFS based on phosphate levels at admission or a drop thereof after starting nutrition.

## Results

104 moderately and severely hypophosphataemic patients were analysed in this study. 84 patients were at risk of refeeding syndrome. 39 (36.5%) patients had a diagnosis of RFS after applying the NICE criteria and initiation of nutrition >10 kcal/kg/day. The mean phosphate levels dropped after admission and reached a nadir on Day 4 (0.69 ± 0.29 mmol/L). Area under the ROC curves (AUC) showed that phosphate levels at admission (AUC 0.51) or a drop of phosphate levels after feed (AUC 0.56) were poor predictors of RFS. The nutritional intake of these patients increased throughout their stay to 1235.5 kcals on day 7, which is lower than recommended intake for adults.

## Conclusions

The incidence of RFS was 36.5%. NICE guidelines for identifying patients at risk for RFS are not applicable to critically ill patients. Electrolytes, especially phosphate levels are weak predictors of RFS. Critically ill patients who were hypophosphataemic were not receiving adequate nutrition during ICU stay. Clinicians should be vigilant for RFS, but must consider alternative causes of hypophosphataemia in critical illness.

